# Telephones for Hospital Use

**Published:** 1913-10-18

**Authors:** 


### TELEPHONES FOR HOSPITAL USE.

### An Automatic Telephone Exchange.

It goes without saying that the telephone should be of great service to hospitals and infirmaries of all kinds. There are a very large number of apparatus upon the market suitable for every kind of institution. For the very smallest institutions there are telephones that can be attached to the ordinary electric-bell service. The bell-push is fitted with two small sockets in the rim of the push, and a double plug, to which a flexible cord is attached, fits into these sockets, the other end of the cord being connected to either a telephone receiver only, or, as is more common now, with the great reduction that has been gradually made in the cost of telephone apparatus, to a combination instrument—a microphone transmitter and telephone receiver connected together by the handle which the user of the instrument grasps. In using this apparatus all that is required is to press the button of the push and remove the combination instrument from a hook, also held by the push, on which it is hung when not in use, and speak in the usual way. This apparatus is only intended for communicating between different parts of the institution, such as the office or kitchen.

An extension of this apparatus, a little more expensive and a little more useful, consists of a push with a bell attached fixed in each room in place of the usual push. A flexible cord with a double plug at one end and a combination instrument at the other completes the arrangement in each room. With this apparatus any room can communicate with any other room by arranging a system of signals. Each room will have its number, and anyone wishing to communicate with any particular room will press the push that number of times and then proceed as before. This apparatus also is only intended for small institutions.

For larger institutions there are various systems, the commonest of which has a complete telephone set in each room or ward to or from which communication is required. Each instrument has what is called a multiple switch attached, a finger that can be moved over a succession of knobs, forming or connected with contact pieces. The knobs are numbered, and all the rooms to which communication is to be made are also numbered, each room having its own knob. The cheapest and commonest form of this apparatus is fixed against the wall. There are other patterns made for standing on the desk; for fixing on a bracket that can be swung out from the wall, and for other positions. These apparatus are made for from five stations, or five rooms, up to twenty. Again, there are several types of the inter-communication telephones, or interphone instruments, as they are sometimes called. With the very simplest form, if, say, 1 and 3 are speaking, and 2 and 4 try to speak, there will be the same confusion as if four people are together in a room, and two of them try to talk together while the other two are also talking, and all are within hearing of each other.

To meet the cases of the larger institutions, where several pairs of rooms, or stations, may require to be speaking together at once, a further development of the apparatus is employed in which a larger number of wires are used. The minimum number of wires required equals the number of stations, plus at least one or two more. To enable more than two stations to communicate together in some forms of apparatus it is claimed that this is accomplished by adding two more wires to the cable in which the whole of the wires are enclosed. For inter-communication telephones all the wires are separately insulated, wrapped with cotton and rubber usually, the whole of them being made up into a cable, which is braided over all. The whole cable is taken to each station, each one of the wires being connected to its own screw terminal upon the instrument in that station. By adding two or three more wires, according to the system employed, it is claimed that two or more pairs of stations can speak together without what is termed "cross talk"—without interfering with each other. On the other hand, this system does not provide for secret conversation, for with the arrangement in which only two or three additional wires are employed anyone taking his telephone off the hook at any station can overhear all the talk that is going on.

A further development that has been introduced to meet the cases where privacy in communication is necessary—what is called the metallic circuit—is employed. That is to say, there are double as many wires in the cable as there are stations, plus at least two other wires. The instruments are more expensive and also there are the additional number of wires in the cable.

For very large institutions a further development is the private telephone exchange. A switchboard is fixed in any convenient position, such as the porter's lodge. The indicator on the switchboard is on the same lines as the ordinary bell indicator that is employed in private houses. It is a more delicate apparatus than the domestic indicator, and is intended partly to fulfil the functions of switching. In the latest modern apparatus the indicator consists of a small incandescent lamp, which lights up when the particular number to which the lamp is connected calls. An attendant is required with this arrangement, and his or her work consists in receiving messages from the different stations and making and unmaking the necessary connections.

The arrangement for making connection between the different stations or rooms calling is very simple. There are flexible cords, with what are called "jacks" at each end. The jack is a special form of plug switch. It does for the exchange switchboard what the double plug described above does for the private domestic telephone, and what the wall plug does for a portable electric light. Each jack carries two conductors, connected to the two wires in the flexible cord, and when the jack is pushed in under the indicator which calls it makes connection with the calling station or room. When the other jack is pushed in under the other indicator connection is made between the calling and the called stations. Removing the jacks leaves both stations ready to receive fresh calls.

### The Automatic Telephone Exchange.

A further development of the telephone exchange system is the automatic telephone exchange, which is steadily making its way. The Postmaster-General, as a recent article in The Hospital showed, is gradually extending its use, and on the Continent of Europe and in America many of the large firms have taken it up. It is employed on the Continent, for instance, by such firms as Krupp's, the great gun and armour-plate people; by the St. George's Hospital at Leipsic; and in this country for the new King's College Hospital. It is used for exchanges numbering as few as twenty, and in private installations up to as many as 800, while public automatic exchanges range from twenty up to 17,000.

In the automatic telephone exchange the work which is done by the operator at an exchange switchboard is performed automatically by a number of electro-magnets. The apparatus fixed in each room or station is very similar to that employed in an ordinary telephone exchange service. It may be fixed on the wall, on the desk, on a hinged bracket, or wherever may be convenient. The only difference is on the front of each instrument there is a disc, having ten finger-holes on one portion of its periphery. Opposite each finger-hole is a number, and the caller counts out the number he requires. Thus, in a hospital, say, of 100 numbers, supposing that No. 56 was required, the caller would place his finger in the hole opposite No. 5, and pull the disc down as far as he was able. He would then release the disc, allowing it to return to its original position; and, placing his finger in the hole opposite 6, would again pull the disc down as far as it went. He would then remove his telephone from the hook, or work the magneto, according to whichever system was in use. The operations which he has carried out, pulling down the disc as far as a stop for his finger will allow him, have sent a succession of electric currents through his line to the automatic electro-magnetic apparatus at the centre. The first current selected the bank of tens in which the number he required was situated, and the second current selected the actual number in that bank. For larger numbers the automatic exchanges are made up to tens of thousands. A similar selection goes on. The first current, for instance, selects the bank of 10,000, the second current selects the bank of 1,000, the third current selects the bank of 100, the fourth current selects the bank of 10, and the fifth current selects the actual number.

If the number he wishes to call is engaged he is automatically apprised of the fact, and in a very few seconds, by a noise in his receiver. When communication is finished the act of replacing the telephones, or the combination instruments, upon their hooks automatically unmakes all the connections that have been made by the first series of operations. The writer suggests that the automatic telephone exchange is very suitable for hospital work. It should save a great deal of time, and as all hospitals now carry their own electrician, there should be no difficulty whatever in the apparatus being kept in order.

As a further extension of the telephone systems described above, some of them—the most expensive—can be arranged to be connected to the town telephone exchange, so that anyone in any part of the institution can communicate with anyone upon the town exchange at will, and, by further switching, with anyone in another town. For this purpose a transfer station, as it is called, is required, and an attendant. The work the attendant has to do is not great. It is merely to receive calls from the different rooms in the institution, call up the exchange, and when the number on the exchange has been obtained, make connection in the usual way.

If communication is required with another town, of course, the usual trunk-line call through the exchange has to be made. The arrangement will be recognised as similar to that which rules in a very large number of modern hotels. One can usually telephone from one's own room to anyone on the exchange in the town where the hotel is situated, and by means of a trunk-line call to anyone in another town. One first calls the attendant in the switch-room in the hotel, she calls the exchange, and so on.

